# OLDER BLUES MUSICIANS

**DOI:** 10.1093/geroni/igz038.2893

**Published:** 2019-11-08

**Authors:** Debra J Sheets

**Affiliations:** University of Victoria, Victoria, British Columbia, Canada

## Abstract

Older female musicians face challenges and advantages from longevity

